# Clinical and Prognostic Relevance of BRIP1 Expression in Colorectal Cancer: Evidence from TCGA and Korean Cohorts

**DOI:** 10.3390/medicina62010047

**Published:** 2025-12-26

**Authors:** Dongbin Park, Yu-Ran Heo, Jae-Ho Lee

**Affiliations:** 1 Medical Course, School of Medicine, Keimyung University, Daegu 42601, Republic of Korea; 2 Department of Anatomy, School of Medicine, Keimyung University, Daegu 42601, Republic of Korea

**Keywords:** BRIP1, colon cancer, rectal cancer, colorectal cancer

## Abstract

*Background and Objectives*: BRCA1-interacting protein C-terminal helicase 1 (BRIP1) encodes a member of the RecQ DEAH helicase family and interacts with the BRCT repeats of breast cancer type 1 (BRCA1). It also participates in DNA damage repair and tumor suppression; thus, its mutations may be associated with an increased risk of several cancers, including fallopian tube and ovarian cancer. Recent research has explored whether BRIP1 dysregulation also contributes to the development and progression of other malignancies. This study investigated the clinical and prognostic value of BRIP1 in colorectal cancer (CRC). *Materials and Methods*: We first analyzed The Cancer Genome Atlas (TCGA) dataset to evaluate the prognostic significance of BRIP1 mRNA expression in CRC. BRIP1 expression was subsequently examined in tumor tissues from 60 CRC patients, and its associations with clinicopathological characteristics and clinical outcomes were assessed. *Results*: In rectal cancer, a higher BRIP1 expression was associated with younger age. In colon cancer, BRIP1 expression was correlated with gender, lymphatic invasion, carcinoembryonic antigen (CEA) level, pathological stage, M stage, N stage, microsatellite instability (MSI) status, and anatomical tumor location. Survival analysis showed that low BRIP1 expression was associated with poorer overall survival in both rectal and colon cancers significantly. In CRC patient tissues, lower BRIP1 expression was further related to elevated CEA levels and unfavorable clinical outcomes. Lower BRIP1 mRNA expression is significantly associated with aggressive clinicopathological features and poor prognosis in CRC. *Conclusions*: BRIP1 may serve as a promising biomarker for risk stratification and a potential therapeutic target in the management of CRC.

## 1. Introduction

Colorectal cancer (CRC) ranked as the third most frequently diagnosed cancer globally in 2022, accounting for 9.6% of newly diagnosed malignancies. It was also the second leading cause of cancer-related death, responsible for 9.3% of global cancer deaths [1]. Both genetic predispositions and lifestyle-related factors—such as consumption of processed foods and high-sugar beverages and obesity—substantially contribute to CRC development [1]. While the majority of CRC cases arise sporadically, 20–30% demonstrate hereditary components, most linked to high-penetrance genetic syndromes, such as Lynch syndrome (3–4%) and familial adenomatous polyposis (around 1%) [2].

The adenoma–carcinoma sequence represents the most widely recognized pathway in CRC tumorigenesis and involves the sequential accumulation of mutations in key regulatory genes such as APC, K-ras, and p53 [3]. Despite extensive research, colorectal tumorigenesis remains incompletely understood, as it results from complex interactions among numerous genes and signaling pathways [4,5]. In particular, dysregulation of DNA damage response (DDR) pathways and genomic instability have been increasingly recognized as important contributors to CRC progression and clinical heterogeneity. Consequently, identifying DDR-related biomarkers that can refine prognostic prediction and potentially guide therapeutic strategies has become an important priority in CRC research.

BRCA1-interacting protein C-terminal helicase 1 (BRIP1), also referred to as FANCJ or BACH1, is encoded by gene located on chromosome 17q22 and belongs to the RecQ DEAH helicase family [6,7]. It interacts directly with BRCA1 and is essential for DNA damage repair and tumor suppression [8]. Alterations in BRIP1 that impair its helicase function have been identified in patients with early-stage breast cancer, and several of these missense variants are considered to confer increased susceptibility to breast cancer [9]. Accordingly, BRIP1 is classified as a moderate-penetrance breast cancer susceptibility gene [10]. Beyond breast cancer, BRIP1 mutations have been implicated in multiple other malignancies, including cervical, ovarian, and prostate cancers [7,9,10,11,12,13,14]. Although BRIP1 has been primarily investigated in the context of germline susceptibility, emerging studies indicate that altered expression of DDR-related genes at the tumor level may influence cancer aggressiveness and patient outcomes, even in sporadic cancers. However, the biological and clinical significance of BRIP1 expression in colorectal cancer remains poorly characterized, and data regarding its prognostic relevance are limited. To address this critical knowledge gap, the present study aimed to systematically evaluate the clinical relevance and prognostic significance of BRIP1 mRNA expression in CRC by integrating transcriptomic data from The Cancer Genome Atlas (TCGA) with analyses of tumor tissues obtained from an independent cohort of 60 CRC patients [15,16]. We hypothesized that reduced BRIP1 expression reflects impaired DDR function in colorectal tumors and is associated with more aggressive clinicopathological features and unfavorable clinical outcomes.

## 2. Materials and Methods

### 2.1. TCGA Data Analysis

**Figure 1:**
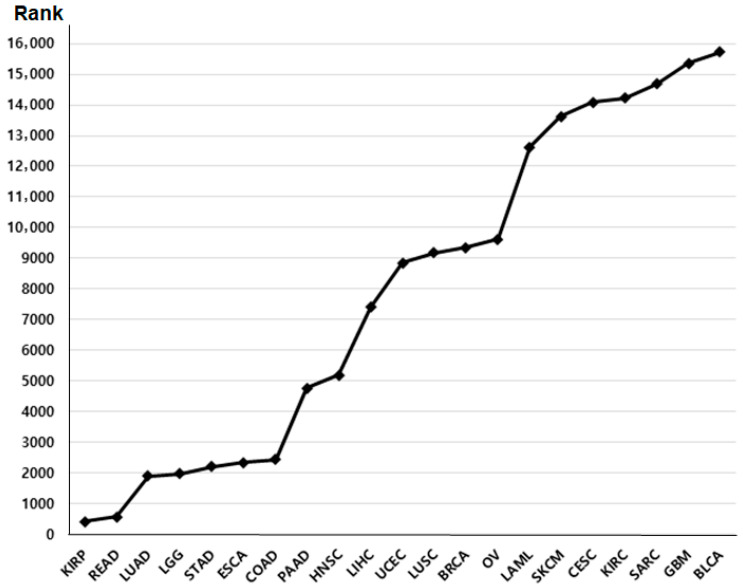
The rank of survival value of BRIP1 in various cancers.

We retrieved primary datasets from The Cancer Genome Atlas (TCGA) portal “http://cancergenome.nih.gov/, accessed on 10 June 2025”. Normalized RNA sequencing (RNA-seq) expression data (TPM values) and corresponding clinical information were obtained from the TCGA-COAD and TCGA-READ cohorts. This database provides *p*-value rankings indicating the prognostic significance of BRIP1 expression across multiple cancer types (Figure 1). Among these cancers, colon and rectal cancers exhibited the most significant associations; therefore, these two cancer types were selected for further in-depth analysis. A total of 440 patients with colon cancer (TCGA-COAD) and 158 patients with rectal cancer (TCGA-READ) were included in the clinical correlation and survival analyses. BRIP1 expression values were log2-transformed for analysis, and patients were stratified into high- and low-expression groups based on the median BRIP1 expression level. Overall survival was defined as the time interval between the date of surgery and the date of death.

### 2.2. CRC Patient Analysis

A total of 60 patients (mean age, 63.6 ± 10.5 years; range, 34–83 years) who underwent colorectal cancer (CRC) surgery at Dongsan Medical Center (Daegu, Republic of Korea) between April 2008 and January 2010 were included in this study. Tumor tissues and paired adjacent non-cancerous tissues were obtained from the Keimyung Human Bioresource Bank. All patients were fully informed of the study purpose, and written informed consent was obtained from each participant. The study protocol was approved by the Institutional Review Board of Keimyung University Dongsan Medical Center (No. 2020-07-027).

Clinicopathological information for all patients was reviewed. Individuals with a history of other malignancies or those who had received preoperative treatments—including chemotherapy, radiofrequency ablation, or transarterial chemoembolization—were excluded. Tumor staging was determined according to the 7th edition of the American Joint Committee on Cancer (AJCC) staging system, based on the characteristics of the primary tumor (T), regional lymph node involvement (N), and distant metastasis (M).

Total RNA was extracted from the tumor and paired non-malignant samples using TRIzol reagent (Molecular Research Center Inc., Cincinnati, OH, USA) [17], and RNA quantity and purity were assessed by measuring absorbance at 260 and 280 nm using a NanoDrop 1000 spectrophotometer (Thermo Scientific, Wilmington, DE, USA). Complementary DNA (cDNA) was synthesized from 2 μg of total RNA using M-MLV reverse transcriptase (Promega, Madison, WI, USA). Quantitative PCR (qPCR) was performed using SYBR Green-based detection with specific primers for BRIP1 as follows: forward 5′-CTTACCCGTCACAGCTTGCTA-3′ and reverse 5′-CACTAAGAGATTGTTGCCATGCT-3′. GAPDH was used as an internal control. RT-qPCR was then carried out on the CFX Connect™ Real-Time PCR Detection System (Bio-Rad, Hercules, CA, USA) using the following program: UNG incubation at 50 °C for 2 min, polymerase activation at 95 °C for 20 s, followed by 40 cycles of denaturation at 95 °C for 30 s and annealing/extension at 60 °C for 30 s. The relative expression level of BRIP1 mRNA was calculated using the 2^−ΔCt^ method, where ∆Ct is the difference between the threshold cycles (Ct) values of the GAPDH and BRIP1 genes. All reactions were performed in triplicate, and five serially diluted control samples were included in each experiment to ensure assay reliability

### 2.3. Statistical Analysis

Statistical analyses were performed using SPSS software (version 25.0; IBM SPSS, Armonk, NY, USA). Patients were stratified into high- and low-expression groups based on the median BRIP1 expression level. Tumor staging was classified according to the 7th edition of the AJCC staging system. Clinicopathological characteristics—including age, sex, carcinoembryonic antigen (CEA) level, and pathological TNM stage—were compared using the chi-square test. Spearman’s correlation analysis was used to evaluate the association between BRIP1 expression and clinical variables in rectal cancer. Univariate survival analysis was conducted using Kaplan–Meier survival curves and the log-rank test. Overall survival was defined as the interval from diagnosis to death. A *p*-value of <0.05 was considered statistically significant.

## 3. Results

### 3.1. BRIP1 Expression in the Cancer Genome Atlas (TCGA) Data

To evaluate the clinical significance of BRIP1 expression, patients with rectal and colon cancer were stratified into high- and low-expression subgroups based on the median BRIP1 expression level (Table 1 and Table 2). In rectal cancer, a higher BRIP1 expression was significantly associated with younger age (59.5% vs. 41.0%, *p* = 0.021). Male sex (55.2% vs. 42.9%), absence of lymphatic invasion (53.7% vs. 40.4%) and absence of venous invasion (52.0% vs. 37.1%), were more frequently observed in the high BRIP1 expression group; however, these associations did not reach statistical significance. No significant correlations were observed between BRIP1 expression and other clinicopathological variables.

In colon cancer, BRIP1 expression demonstrated significant associations with female (55.6% vs. 45.3%, *p* = 0.031), absence of lymphatic invasion (56.0% vs. 40.8%, *p* = 0.003), low CEA level (57.1% vs. 42.2%, *p* = 0.020), lower pathological stage (*p* = 0.022), no M stage (50.8% vs. 36.1%, *p* = 0.035), lower N stage (*p* = 0.002), microsatellite instability (MSI) status (*p* < 0.001), and anatomic tumor location (*p* = 0.030).

**Figure 2:**
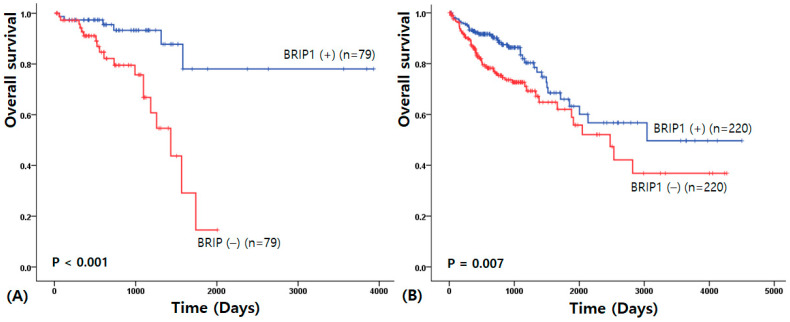
Overall survival analysis for rectal (**A**) and colon (**B**) cancer.

Overall survival analysis indicated that BRIP1 expression had significant prognostic value in both rectal and colon cancer. In rectal cancer, patients with high BRIP1 expression exhibited markedly longer overall survival compared with those with low expression (3324.42 ± 269.84 vs. 1305.64 ± 98.40 days; χ^2^ = 13.94; *p* < 0.001) (Figure 2A). Similarly, in colon cancer, high BRIP1 expression was associated with improved survival outcomes (2975.55 ± 222.49 vs. 2421.43 ± 206.15 days; χ^2^ = 7.304; *p* = 0.007) (Figure 2B).

### 3.2. BRIP1 Expression in CRC Patients

BRIP1 expression was analyzed in 60 CRC tissues, and its expression level was not significantly different between tumor and paired adjacent non-tumorous tissues (*p* = 0.57). When normalized as the tumor-to-non-tumor expression ratio, the mean BRIP1 expression level was 2.71 ± 1.62.

To identify the clinicopathological significance of BRIP1, patients were stratified into high- and low-expression groups based on the median BRIP1 expression value. The clinicopathological characteristics associated with BRIP1 mRNA expression in CRC patient tissues are summarized in Table 3. Higher BRIP1 expression was significantly associated with lower carcinoembryonic antigen (CEA) levels (57.1% vs. 42.2%, *p* = 0.028), whereas no other clinicopathological variables showed statistically significant associations.

**Figure 3:**
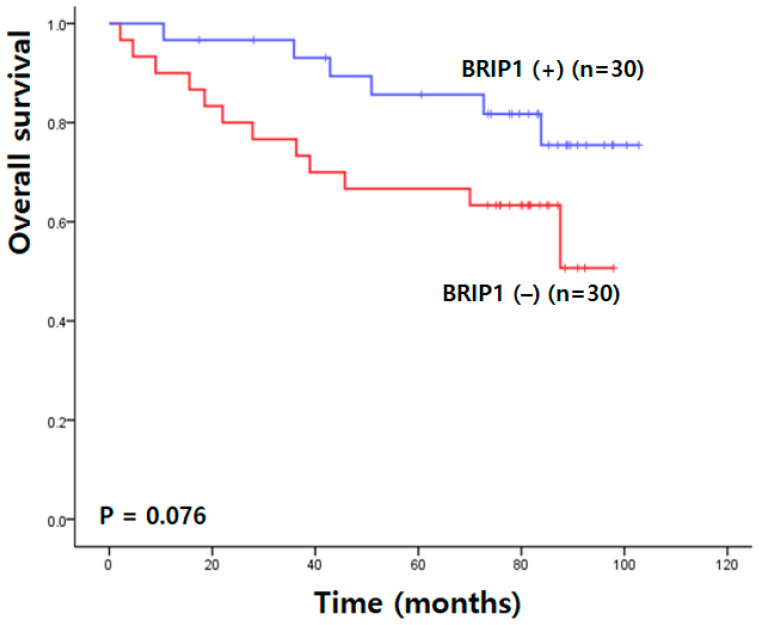
Overall survival analysis in Korean patients with colorectal cancer.

Kaplan–Meier survival analysis demonstrated that patients with higher BRIP1 expression tended to have improved overall survival compared with those with lower expression; however, this difference did not reach statistical significance (90.80 ± 4.65 vs. 70.40 ± 6.58 months; χ^2^ = 3.14; *p* = 0.076) (Figure 3).

## 4. Discussion

In this study, we evaluated the clinical relevance and potential prognostic association of BRIP1 expression in colorectal cancer using TCGA datasets and an independent cohort of 60 CRC patients. The primary objective of this study was to investigate the clinical significance of BRIP1 at the mRNA expression level by directly comparing large-scale transcriptomic data with real-world patient samples. To our knowledge, this is the first study to comprehensively examine BRIP1 expression in both rectal and colon cancers and to integrate RNA expression profiling from patient tissues.

As revealed through TCGA data, a higher BRIP1 expression was significantly associated with younger age in rectal cancer. In colon cancer, BRIP1 expression showed significant associations with several clinicopathological parameters, including gender, lymphatic invasion, CEA levels, pathological stage, M stage, N stage, MSI status, and anatomic tumor location. Kaplan–Meier survival analyses demonstrated that lower BRIP1 expression was associated with poorer overall survival in both colon and rectal cancers, supporting a prognostic association. Importantly, these findings suggest a correlational rather than a strictly independent prognostic role, indicating that BRIP1 expression may reflect underlying tumor biology rather than act as a standalone prognostic determinant. In our CRC patient cohort, BRIP1 mRNA levels were significantly associated with the serum CEA level, although other clinicopathological variables did not exhibit statistically significant relationships. Although higher BRIP1 expression tended to be associated with improved overall survival, this trend did not reach statistical significance—likely due to the small sample size. Nonetheless, the directionality of survival trends was consistent with TCGA findings, supporting the hypothesis that reduced BRIP1 expression may be associated with more aggressive tumor behavior. Taken together, these findings suggest that transcript-level alterations of BRIP1 alone may capture clinically meaningful prognostic information in colorectal cancer.

From a biological perspective, BRIP1 has emerged as a key component of the DNA damage response (DDR) machinery, functioning as a helicase that cooperates with BRCA1 in homologous recombination-mediated DNA repair. Dysregulation of BRIP1 can lead to genomic instability, a hallmark of colorectal cancer progression [18]. Recent genomic and transcriptomic studies have reported BRIP1 mutations or reduced expression in subsets of sporadic CRC, particularly those exhibiting defective homologous recombination or chromosomal instability signatures [19]. These observations support the concept that BRIP1 may influence colorectal tumor biology not only through genetic alterations, but also through transcriptional dysregulation detectable at the mRNA level. Collectively, these findings position BRIP1 mRNA expression as a biologically relevant and clinically informative molecular feature, while also providing a rationale for future integrative multi-omics investigations.

This study has several limitations. First, the analyzed TCGA cohort was relatively small compared to other cancer datasets, underscoring the need for larger, multi-institutional studies with multivariate analyses. Second, although this study intentionally focused on mRNA expression to ensure consistency and comparability across large datasets and patient cohorts, transcriptomic data may not fully reflect BRIP1 protein abundance or functional activity due to post-transcriptional or post-translational regulation. Future validation at the protein level, using immunohistochemistry, Western blotting, or proteomic approaches, will therefore be essential. Third, while our findings suggest a prognostic association, functional studies are required to elucidate the mechanistic role of BRIP1 in colorectal cancer progression. Such studies should include in vitro and in vivo models involving BRIP1 knockdown or overexpression, DNA repair capacity assays, and pathway-based analyses.

**Figure 4:**
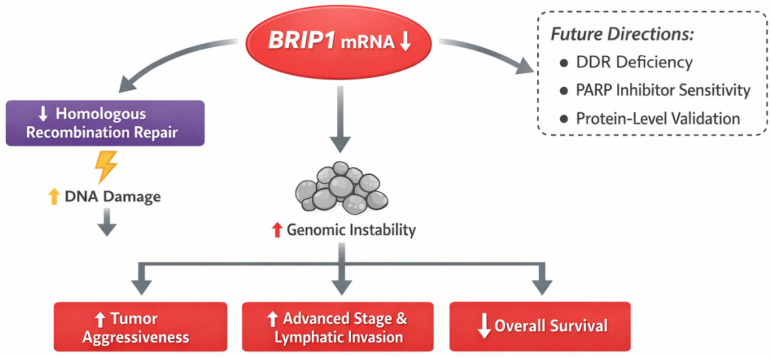
Overview of BRIP1 Expression in Colorectal Cancer and Future Research Directions.

Finally, future investigations integrating genomic alterations, MSI status, proteomic profiles, and therapeutic response data may clarify whether BRIP1 serves as a surrogate marker of DDR deficiency and a potential predictor of response to DNA damage-targeted therapies, such as PARP inhibitors (Figure 4). Such integrative approaches may ultimately establish BRIP1 as a clinically actionable biomarker in colorectal cancer.

## 5. Conclusions

This study demonstrated that BRIP1 expression is significantly associated with key clinicopathological features and overall survival in colorectal cancer based on TCGA analyses. These findings were further supported by the BRIP1 expression patterns observed in patient tumor tissues. Collectively, our results suggest that BRIP1 expression may have potential prognostic relevance in colorectal cancer; however, these findings should be interpreted as exploratory. Additional studies integrating larger clinical cohorts, comprehensive protein-level validation, and mechanistic experiments are required to elucidate the biological roles of BRIP1 and to determine its applicability in clinical decision-making and therapeutic development.

## Figures and Tables

**Table 1:** Clinical characteristics of BRIP1 expression in rectal cancer for TCGA data.

	BRIP1		
	High (*n*, %)	Low (*n*, %)	*p*-Value
Age			
<65	44 (59.5)	30 (40.5)	0.021
≥65	34 (41.0)	49 (59.0)	
Gender			
Female	30 (42.9)	40 (57.1)	0.125
Male	48 (55.2)	39 (44.8)	
Lymphatic invasion			
No	44 (53.7)	38 (46.3)	0.122
Yes	23 (40.4)	34 (59.6)	
CEA			
≤5	35 (56.5)	27 (43.5)	0.863
>5	25 (58.1)	18 (41.9)	
Venous invasion			
No	53 (52.0)	49 (48.0)	0.130
Yes	13 (37.1)	22 (62.9)	
Pathological stage			
Stage I	15 (51.7)	14 (48.3)	0.403
Stage II	24 (51.1)	23 (48.9)	
Stage III	23 (48.0)	25 (52.0)	
Stage IV	10 (41.7)	14 (58.3)	
M stage			
M0	59 (50.0)	59 (50.0)	0.849
M1	11 (47.8)	12 (52.2)	
N stage			
N0	41 (51.9)	38 (48.1)	0.434
N1	21 (48.8)	22 (51.2)	
N2	14 (43.8)	18 (56.2)	
T stage			
T1	5 (55.6)	4 (44.4)	0.129
T2	13 (48.1)	14 (51.9)	
T3	56 (52.3)	51 (47.7)	
T4	4 (30.8)	9 (69.2)	

**Table 2:** Clinical characteristics of BRIP1 expression in colon cancer for TCGA data.

	BRIP1		
	High (*n*, %)	Low (*n*, %)	*p*-Value
Age			
<65	80 (47.1)	90 (52.9)	0.309
≥65	140 (52.0)	129 (48.0)	
Gender			
Female	114 (55.6)	91 (44.4)	0.031
Male	106 (45.3)	128 (54.7)	
Lymphatic invasion			
No	136 (56.0)	107 (44.0)	0.003
Yes	62 (40.8)	90 (59.2)	
CEA			
≤5	108 (57.1)	81 (42.9)	0.020
>5	38 (42.2)	52 (57.8)	
Venous invasion			
No	152 (52.4)	138 (47.6)	0.256
Yes	41 (46.0)	49 (54.0)	
Pathological stage			
Stage I	44 (61.1)	28 (38.9)	0.022
Stage II	90 (52.9)	80 (47.1)	
Stage III	58 (46.0)	68 (54.0)	
Stage IV	22 (36.0)	39 (64.0)	
M stage			
M0	165 (50.8)	160 (49.2)	0.035
M1	22 (36.1)	39 (63.9)	
N stage			
N0	145 (56.4)	112 (43.6)	0.002
N1	48 (46.2)	56 (53.8)	
N2	27 (34.6)	51 (65.4)	
T stage			
T1	7 (63.6)	4 (36.4)	0.222
T2	43 (58.1)	31 (41.9)	
T3	149 (49.3)	153 (50.7)	
T4	21 (41.2)	30 (58.8)	
Histological type			
Colon adenocarcinoma	187 (50.0)	187 (50.0)	0.811
Colon mucinous adenocarcinoma	31 (51.7)	29 (48.3)	
MSI			
Indeterminate	2 (100.0)	0 (0.0)	<0.001
MSI-H	55 (72.4)	21 (27.6)	
MSI-L	36 (45.6)	43 (54.4)	
MSS	123 (44.9)	151 (55.1)	
Colon polyps			
No	130 (53.7)	112 (46.3)	0.211
Yes	61 (46.9)	69 (53.1)	
Anatomic neoplasm			
Ascending colon	41 (54.3)	44 (51.8)	0.030
Cecum	49 (48.5)	52 (51.5)	
Descending colon	7 (36.8)	12 (63.2)	
Hepatic flexure	16 (59.3)	11 (40.7)	
Rectosigmoid Jun	1 (100.0)	0 (0.0)	
Sigmoid colon	73 (50.3)	72 (49.7)	
Splenic flexure	5 (71.4)	2 (28.6)	
Transverse colon	19 (50.0)	19 (50.0)	

**Table 3:** Clinical characteristics of BRIP1 expression in Korean patients with colorectal cancer.

	BRIP1		
	High (N, %)	Low (N, %)	*p*-Value
Age			
<65	15 (47.1)	10 (52.9)	0.190
≥65	15 (52.0)	20 (48.0)	
Lymphatic invasion			
No	12 (56.0)	14 (44.0)	0.602
Yes	18 (40.8)	16 (59.2)	
CEA			
≤5	27 (57.1)	20 (42.9)	0.028
>5	3 (42.2)	10 (57.8)	
Pathological stage			
Stage I/II 11	7 (61.1)	4 (38.9)	0.317
Stage III/IV 49	23 (52.9)	26 (47.1)	
M stage			
M0	29 (50.8)	27 (49.2)	0.301
M1	1 (36.1)	3 (63.9)	
N stage			
N0	19 (56.4)	14 (43.6)	0.194
N1/N2	11 (46.2)	16 (53.8)	
T stage			
T1/T2	6 (63.6)	5 (36.4)	0.739
T3/T4	24 (49.3)	25 (50.7)	
Differentiation			
Good/Moderate	29 (50.0)	27 (50.0)	0.301
Poor	1 (50.0)	3 (50.0)	

## Data Availability

The datasets used and/or analyzed during the current study are available from the corresponding author.
